# The Role of Transformational Leadership and Knowledge Management and Learning Organization on Vocational Schools Performance During Digital Era

**DOI:** 10.3389/fpsyg.2022.895341

**Published:** 2022-05-06

**Authors:** Arif Firmansyah, Ming-Huei Chen, I Wayan Ruspendi Junaedi, Mokhammad Arwani, Anang Kistyanto

**Affiliations:** ^1^Department of Management, Universitas Airlangga, Surabaya, Indonesia; ^2^GITM, National Chung Hsing University, Taichung, Taiwan; ^3^Department of Management, Universitas Dhyana Pura Bali, Bali, Indonesia; ^4^Department of Management, Universitas Wahid Hasyim, Semarang, Indonesia; ^5^Department of Management, Universitas Negeri Surabaya, Surabaya, Indonesia

**Keywords:** transformational leadership, knowledge management, learning organization, schools performance, digital era

## Introduction

In the digital era and the industrial revolution 4.0, one of the problems of vocational schools is the low production of graduates with low skills. Graduates with low skills are difficult to hire due to strict recruitment requirements (Birasnav et al., 2013). This resulted in an increase in the unemployment rate. The high unemployment rate is probably caused by the low quality of vocational school graduates. This is the output of the low quality of campus management. They were not recruited because they did not meet the company's recruitment criteria. To overcome this problem, campus management needs to achieve efficiency and effectiveness, create employee engagement programs and lecturer training, and ensure lecturer job satisfaction (Vizano et al., 2020). There have been several previous studies on the effect of transformational leadership and knowledge management on organizational performance. Transformational leadership has a positive effect on organizational performance and some explain that the relationship between transformational leadership and organizational performance is mediated by a variable Knowledge management has a positive effect on organizational performance (Vizano et al., 2020; Suharto et al., 2022). Several studies by Kadiyono et al. (2020) and Purwanto et al. (2021a,b) explains that knowledge management and organizational performance relationship are mediated by variables. In general, previous studies used the leadership of profit-oriented business entities as the unit of analysis. In other words, it is still rare for previous studies to use the chancellor of private universities as the unit of research analysis so far. As a result, the influence of transformational leadership and knowledge management as independent variables and learning organization as a mediating variable on the organizational performance of vocational schools as dependent variables in the context of vocational schools is rarely discussed in the literature. In examining the concept of leadership, there are two complementary approaches; namely physiological aspects and psychological aspects. The first focuses more on things that are concrete and rational; while the second, more toward the emotional dimension. To get a comprehensive understanding of leadership, each of these approaches must have a balanced portion. On this limited opportunity, this paper limits itself to the study of the psychological aspect and management. To achieve this goal, several steps were taken. It is hoped that this research theoretically contributes in the field of psychological aspect and strategic management. Practically, vocational school leaders are able to apply research results to improve the quality of campus management. This study makes two contributions to the literature. This study describes the implementation of transformational leadership, knowledge management, organizational learning, and organizational performance.

## Transformational Leadership and Learning Organizations

Transformational Leadership and Learning Organizations Transformational leadership is a form of leadership that can push followers to higher levels of performance (Vizano et al., 2020; Suharto et al., 2022). It is the practice of identifying the motivations, values, and needs of superiors and subordinates with the aim of satisfying the whole group. And, it focuses on developing followers and their needs by means of increasing teacher engagement, commitment and performance. Transformational leadership is the art of motivating subordinates to achieve the highest performance in doing their jobs and contributing to the success of their organizations by developing their competencies and meeting their needs (Degrees et al., 2014; Kadiyono et al., 2020; Haudi et al., 2022). Some researchers (Quddus et al., 2020; Sunarsi et al., 2020; Purwanto et al., 2021a,b) have proven the influence of transformational leadership on learning organizations.

## Knowledge Management and Learning Organization

Knowledge management is defined as the processes and activities that help organizations generate, acquire, discover, and organize knowledge and use and disseminate it among the people who work, and transfer the information and experience that the organization has and apply it in its management activities, such as decision making, decisions, work procedures, and strategic planning (Quddus et al., 2020; Sunarsi et al., 2020). It is a company's strategic ability to acquire knowledge, manage and utilize market knowledge. Knowledge management is the transfer of important information and expertise as part of organizational memory and its nature in the organization indirectly structured into structured knowledge in order to achieve organizational goals. Based on the above definition, it can be concluded that management Knowledge is the process of acquiring and disseminating knowledge to lecturers within an organization in an attempt to leverage it to find solutions of the problems facing the organization. Some researchers (Quddus et al., 2020; Vizano et al., 2020; Suharto et al., 2022) have proven the effects of knowledge management on learning organizations.

## Organizational Learning and Organizational Performance

As previously explained, a learning organization is an organization that provides the widest opportunity to develop one's potential through learning and sharing knowledge together. Several researchers have proven the effect of organizational learning on organizational performance. Organizational performance is a complex and multidimensional phenomenon in the business literature. It can be most simply defined as the company's performance compared to its goals and objectives. It consists of results organization or the actual output of an organization, which can be measured against the output, desired goals and objectives Indicators to measure organizational performance are agreement that all employees are satisfied with working at the university; and the agreement that all lecturers use resources efficiently in doing their work.

From some of the results of these thoughts and also supported by the findings previous research (Vizano et al., 2020; Suharto et al., 2022) the following hypotheses can be made.

## Method

**Figure 1:**
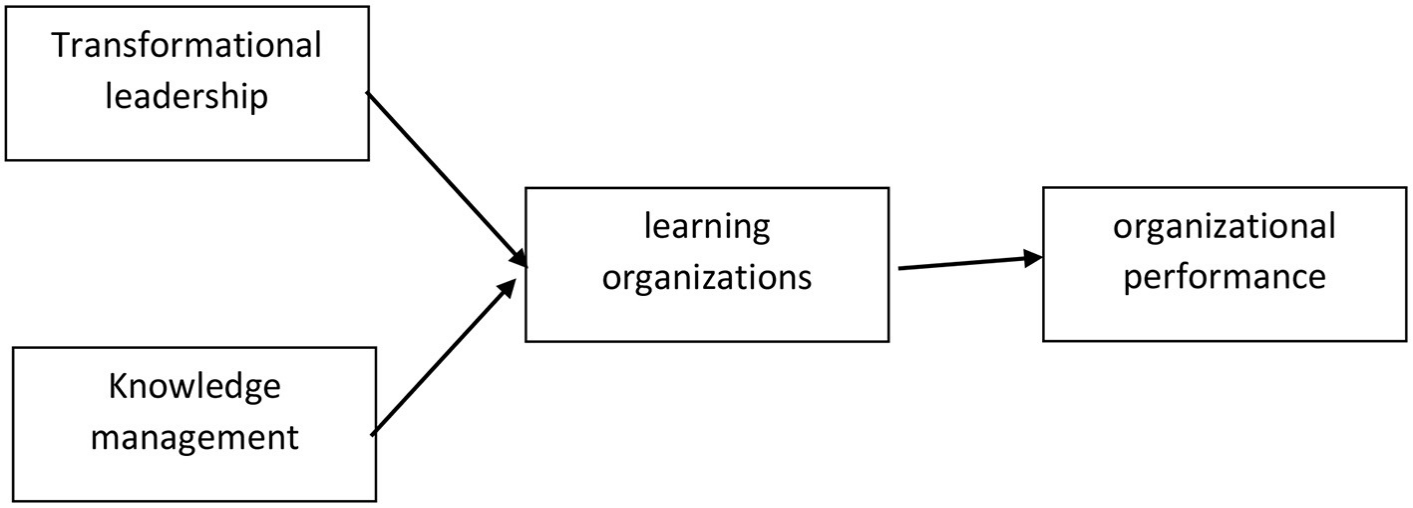
Theoretical framework.

This research uses quantitative methods. The data were analyzed using the Partial Least Square (PLS) method with the Smart PLS program. This study describes the measured variables and examines the relationship between variables such as the relationship between transformational leadership and knowledge management on the organizational performance of vocational schools mediated by learning organizations. The independent variables of this research are transformational leadership and knowledge management. The dependent variable is organizational performance while the intervening variable is learning organization as per Figure 1. Respondents are leaders of vocational schools. The reason for making vocational school leaders to become respondents is because they have experienced and implemented all of them measured variable. There were 20 respondents who were selected by simple random sampling method. Questionnaires were sent online to 150 leaders at random. A total of 120 leaders participated as respondents. Indicators to measure knowledge management in this study are agreement that universities use storage such as online repositories to store scientific writings from lecturers and students, agreement that universities use various sources to obtain knowledge, and agreement that universities facilitate lecturers' tacit knowledge media into documented writings good for supporting the implementation of knowledge management.

## Discussion

Based on the results of the analysis that the direction of the relationship between management knowledge and learning organization is positive and significant, the direction of the relationship between transformational leadership and organizational learning is positive, and the direction of the relationship between organizational learning and organizational performance is positive. Overall, the results show that transformational leadership has a positive effect on learning organizations. This means that all leaders in vocational schools are required to communicate and discuss openly. This open discussion among leaders will encourage all lecturers to share knowledge and skillsthem and make lecturers want to learn new things related to their work and career. This is a good moment for leaders to push subordinates to learn more about learning strategies and facilitate subordinates with continuous training programs. This finding confirms the previous study conducted by Crawford (2003), Birasnav et al. (2013), Gelard et al. (2014), Desky et al. (2020), Kadiyono et al. (2020), Purwanto et al. (2021a,b), and Haudi et al. (2022).

Knowledge management also affects learning organizations. This implies that university leaders provide all lecturers with an online repository for enable lecturers to access knowledge in e-books and e-journals and expand their knowledge. In addition, the leadership also provides a system and structure for classifying science and knowledge so that lecturers can easily and quickly study documents. The leaders also encourage all lecturers to write their tacit knowledge to share experience with other lecturers through written documents that they produce and archive in the repository. Repositories, systems and structures of knowledge and science classification and documentation of tacit knowledge will motivate lecturers to enrich their learning strategies and take opportunities to learn. This research confirms research by Quddus et al. (2020), Sunarsi et al. (2020), Vizano et al. (2020), Purwanto et al. (2021a,b), and Suharto et al. (2022).

The discussion of the psychological aspects of leadership is a small part of the study of leadership psychology. Therefore, there are at least three things that need to be understood in this paper, first, the field of psychology is much broader and more complicated than the study of its psychological aspects, which therefore requires quite a large amount of time to get a complete and in-depth understanding, second, the field of leadership psychology is a relatively more “abstract” study than its psychological aspect which shows its concrete form, although it is a bit vague, in real behavior, and third, for the beginner stage (for those who are new to the discipline of psychology) it is very good to study the concept of leadership starting from a more specific field. In Leadership, the psychological aspect refers to how a leader is able to be a role model for his subordinates, so that what he wants (in the context of the organization) is followed, everything he is ordered to do as well as possible, and anything he forbids is obeyed to be avoided. Exemplary is realized because he has certain advantages that his subordinates do not have. Among the advantages that can lead a leader to be an example for his subordinates are his superiority in terms of personal integrity, mastery of science and technology, aspiration, appreciation, quick decision making and taking action, and the like. The description of the psychological aspect is generally accepted in solid organizations, including in professional educational institutions.

## Conclusion

The main finding in this study is that the implementation of transformational leadership, knowledge management, and learning organization has been carried out very well in vocational schools. The implementation of vocational school organizational performance is well implemented and still needs to be improved. The second finding is that there is a positive effect of transformational leadership and knowledge management on learning organizations. It was also found that organizational learning affects organizational performance and mediates the relationship between transformational leadership and knowledge management on vocational school performance. From the relationship between these variables, it can be interpreted that to improve organizational performance, leadership private universities need to encourage all lecturers and lecturers to develop strategies learning and provide learning opportunities for lecturers. In addition, the leader the university also develops open discussion and communication among lecturers so that lecturers can share knowledge and skills to solve the problems they face in doing their work. Furthermore, this research only looks at the relationship between the independent variable and the dependent variable mediated by the mediator. Of course, there is still the possibility of a direct relationship between the independent variable and the dependent variable. Furthermore, there are other independent variables that have the possibility to affect the performance of vocational schools. Being a leader needs basic capital to be able to carry out managerial functions. Among the main and basic managerial functions that a leader needs to have is the transformational leadership model.

## Author Contributions

AF contributed to conceptualization, methodology, investigation, curation, analysis, funding acquisition, and writing. M-HC helped in investigation, curation, analysis, and writing. IJ, MA, and AK helped in review, analysis, and writing. All authors contributed to the article and approved the submitted version.

## Conflict of Interest

The authors declare that the research was conducted in the absence of any commercial or financial relationships that could be construed as a potential conflict of interest.

## Publisher's Note

All claims expressed in this article are solely those of the authors and do not necessarily represent those of their affiliated organizations, or those of the publisher, the editors and the reviewers. Any product that may be evaluated in this article, or claim that may be made by its manufacturer, is not guaranteed or endorsed by the publisher.
